# Impact of a pharmacy-driven MRSA nares screening protocol on vancomycin discontinuation in a tele-antimicrobial stewardship model

**DOI:** 10.1017/ash.2024.43

**Published:** 2024-04-22

**Authors:** Alex M. Trzebucki, Matthew R. Davis, Erin K. McCreary, Susan Cuccaro, J. Ryan Bariola, Tina Khadem

**Affiliations:** 1 Division of Infectious Diseases, Department of Medicine, University of Pittsburgh School of Medicine, Pittsburgh, PA, USA; 2 Department of Clinical Pharmacy, Infectious Disease Connect, Inc., Pittsburgh, PA, USA; 3 Department of Clinical Pharmacy, Heritage Valley Health System, Beaver, PA, USA

## Abstract

A pharmacist-driven protocol for methicillin-resistant *Staphylococcus aureus* nares screening and empiric vancomycin discontinuation was instituted in a community healthcare system utilizing a tele-antimicrobial stewardship program to reduce inappropriate use of vancomycin. The protocol and associated intervention resulted in a significant decrease in both vancomycin utilization and the rate of acute kidney injury.

## Introduction

Vancomycin is often initiated empirically for patients admitted for pneumonia despite the low prevalence of methicillin-resistant *Staphylococcus aureus* (MRSA) as the causative pathogen in patients hospitalized with pneumonia.^1^ Published literature has demonstrated the utility of MRSA nasal screening assays to discontinue empiric vancomycin for pneumonia in low-prevalence communities. These data are predominantly validated in academic centers that feature on-site antimicrobial stewardship programs (ASP).^2,3^ Despite this, antimicrobial utilization in the community setting often matches or exceeds utilization in academic hospitals.^4^ The challenges of achieving stewardship goals in a community hospital are unique due to limited resources, unfamiliarity with stewardship practices, and lack of infectious disease (ID) trained experts.^5–7^ Tele-antimicrobial stewardship (TASP) is a novel means of bringing ID and ASP expertise to community non-academic hospital settings.^8^ To our knowledge, the role of MRSA nares screening in a TASP model has not been described.

Herein, we highlight the implementation and result of a TASP-designed protocol for non-ID trained clinical pharmacists to assess, screen, and discontinue vancomycin utilizing MRSA nares testing via culture-based and polymerase chain reaction (PCR)-based methodologies. We discuss how the protocol affected vancomycin usage and patient outcomes in the community hospital setting.

## Methods

Implementation occurred at a 3-hospital, nonteaching, community healthcare system in Western Pennsylvania that encompasses a total of 585 licensed beds with an average case mix index of 1.71. The system utilized a TASP via a hub-and-spoke model with central ID experts advising local non-ID clinical pharmacists assigned to dedicated stewardship coverage. The TASP utilized prior authorization for select antimicrobials and daily prospective audit with intervention and feedback via synchronous ASP calls between the local pharmacists and central ID team (calls occurred Monday through Friday). All interventions were made and tracked by the local stewardship pharmacists. Baseline antimicrobial use data suggested elevated use of vancomycin with pneumonia listed as a frequent indication. Existing MRSA nares testing relied on chromogenic agar (CA) culture-based assay, and no stewardship protocol existed to guide and oversee MRSA nares testing or vancomycin discontinuation based on testing results.

A pharmacy protocol was authored by the central tele-ID team alongside local pharmacy, medical, and microbiology leadership. The protocol was approved by hospital leadership as an Instituitional Review Board-exempt quality improvement project and went live on April 1, 2021. The intervention period was divided into two phases where the protocol first utilized the existing CA culture-based (CHROMagar™ MRSA) MRSA nares assay (phase I: 4/2021–9/2021) followed by the transition to a PCR-based assay (Cepheid GeneXpert®) (phase II: 4/2022–9/2022) to assess the incremental impact of rapid molecular testing on TASP protocol effectiveness. Intervention periods (phases I and II) were compared with a preintervention period (4/2019–9/2019) where the CA-based MRSA nares assay was available for providers to order without antimicrobial stewardship oversight.

**Figure 1.**
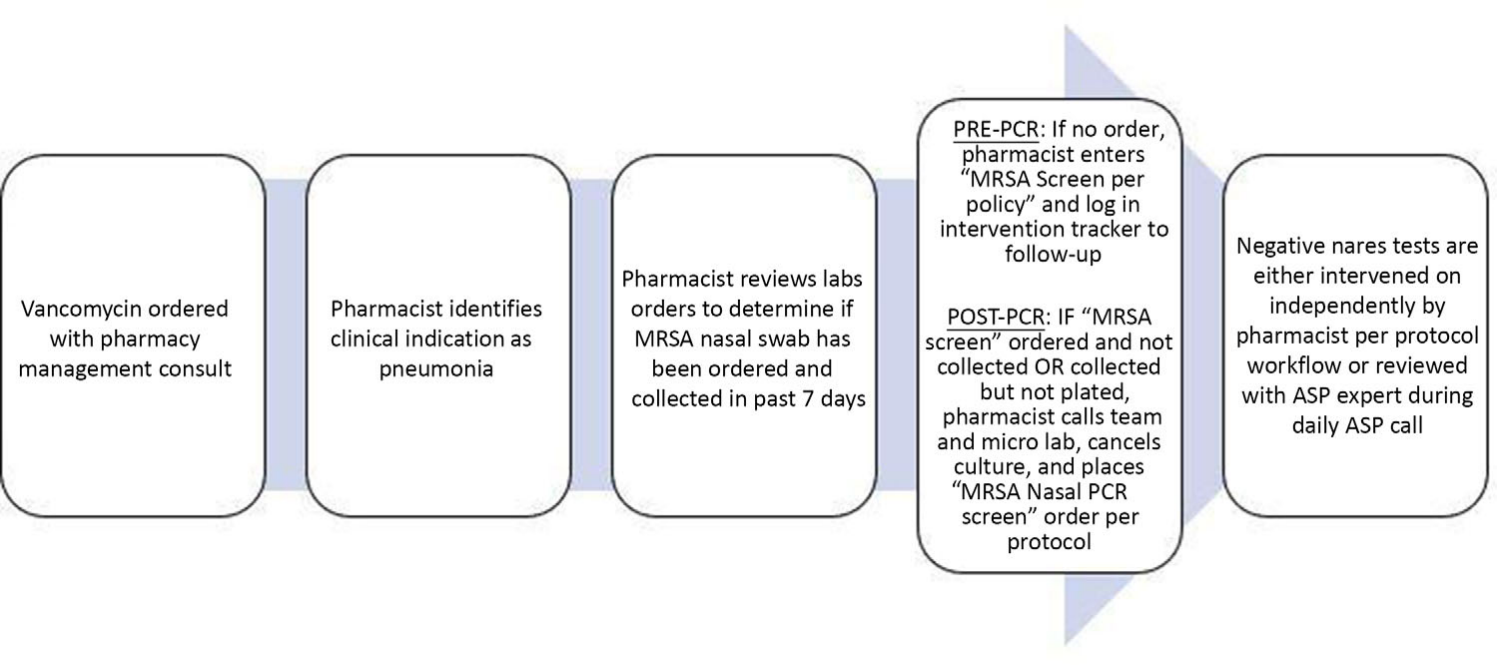
Pharmacist MRSA nares protocol workflow. *Note*: MRSA, methicillin-resistant *Staphylococcus aureus*.

Pharmacists reviewed all patients admitted to one of the three system hospitals who had vancomycin ordered with the indication of either community-acquired pneumonia (CAP) or hospital-acquired pneumonia/ventilator-associated pneumonia (HAP/VAP) (Figure 1). MRSA nares testing was ordered by the local pharmacists per protocol. The pharmacist communicated with the primary nurse to ensure correct specimen collection. The microbiology laboratory batched MRSA CA cultures once daily in the morning which resulted after 24 hours. Upon transition to the PCR-based assay, samples were run as received during regular daytime hours with a test turnaround time of 1 hour. Assay results were available in the electronic health record (EHR) with a stewardship nudge addressing the negative predictive value of a negative test result in pneumonia. Pharmacists screened assay results using either the EHR or antimicrobial stewardship software (TheraDoc®, Charlotte, NC) once daily. Negative test results were verbally communicated to the primary medical team. Antimicrobial stewardship guidance to discontinue empiric vancomycin was provided when no other appropriate indication for vancomycin therapy was present as per protocol criteria (Supplemental Figure 1). All local pharmacists and primary providers received initial in-person and newsletter education on the evidence basis for MRSA nares testing, testing indication, protocol workflow, and exclusion criteria from nares-based vancomycin de-escalation.

Interventions were tracked in an antimicrobial stewardship intervention tracker, and manual data extraction was performed from the EHR. Outcome measures included the rate of MRSA swabs ordered, efficiency metrics (time to swab order, collection, and result), duration of vancomycin therapy among patients with negative MRSA screen results, rate of same-day vancomycin discontinuation, rate of  acute kidney injury (AKI) (serum creatinine elevation of ≥ 0.3mg/dl from baseline per Kidney Disease Improving Global Outcomes guidelines), and rate of nares to respiratory culture discordance.^9^ MRSA nares test characteristics were evaluated against respiratory cultures collected within 7 days of screening.

Data were summarized using descriptive statistics including means, frequencies, and percentages. Bivariate analyses were conducted using χ^2^ or Fisher’s Exact for categorical variables and the Mann-Whitney test for non-normally distributed continuous variables. All statistical analyses were performed in SAS statistical software, version 9.4 (SAS Institute, Cary, North Carolina).

## Results

Six hundred and twenty-eight patients were included, 249 in the preintervention period, 170 in the CA protocol period, and 209 in the PCR protocol period. The mean age was 70.3 years, 51% male, and 91% Caucasian in the preintervention period and 68.8 years, 55% male, and 86% Caucasian in the intervention period. Upon the institution of the pharmacy MRSA nares protocol, MRSA swabs ordered for the indication of CAP or HAP/VAP increased from 65.5% to 95.3% (*P* < .0001) with 75% of swabs ordered by pharmacists (Supplemental Table 1). The mean time to swab collection decreased from 2 days after vancomycin initiation to the same day of vancomycin initiation (collection: 1.5 d vs 0.7 d, *P* < .0001). The time to swab result decreased by 66%, from 2 days to the same day (1.8 d vs 0.2 d, *P* <.0001) with the PCR assay.

Among patients with a negative MRSA screen, vancomycin days of therapy decreased with the greatest effect noted upon the implementation of the PCR assay where a 50% decrease in duration, or 2 full days, was noted (Table 1). The rate of same-day vancomycin discontinuation increased with the greatest effect noted after PCR assay was instituted. Rates of AKI decreased 13% in the postintervention period (25.3% vs 12.0%, *P* = .0005). Rates of MRSA nares-negative and respiratory culture-positive discordant results were noted at 0%–1.9% throughout the study periods. The evaluation of test characteristics was performed during the CA phase and demonstrated a positive predictive value of 37.5% and negative predictive value of 95.83% (Supplemental Table 2).

**Table 1.** Outcome measures across intervention periods

	Preintervention (n = 249)	Postintervention CA (n = 170)	Postintervention PCR (n = 209)	*P* value
Duration of vancomycin among negative screens (days—mean)	3.6	3.1	2.0	< .001
Same-day discontinuation (percent)	20.1%	32.2%	36.1%	< .0001
Discontinuation within one day of result (percent)	52.1%	69.8%	64.3%	.0181
AKI (percent)	25.3%	14.8%	12.0%	.0005

Note. CA, chromogenic agar; PCR, polymerase chain reaction. AKI

## Discussion

We demonstrate the implementation and impact of a pharmacy-driven MRSA nares protocol at a multi-hospital, nonteaching, community health system utilizing a TASP model. The protocol empowered local pharmacists assigned to stewardship service to screen all hospitalized patients for the appropriateness of MRSA nares assay and manage the testing and de-escalation workflow with a clinical guidance document and TASP oversight where appropriate. The protocol implementation increased appropriate MRSA nares screening, decreased the duration of antibiotic therapy by one day, and improved the rate of same-day vancomycin discontinuation. Further, rates of AKI dropped significantly. Programmatic success was driven by the rapid turnaround of the PCR assay allowing pharmacists to implement stewardship intervention during normal work hours. Our implementation highlighted that establishing an effective program for de-escalating empiric vancomycin therapy in a TASP model requires utilization of rapid MRSA testing and a collaboratively designed protocol.

The retrospective nature and lack of a nonintervention control group are key limitations of our study. As such the descriptive pre- and postintervention analysis utilized with historic controls to compare our interventions may introduce bias. Changes in outcome measures from the historical control to the intervention periods could be explained by other unmeasured factors or preexisting trends in declining vancomycin use. Additionally, the use of a preintervention period prior to the coronavirus disease 2019 pandemic may introduce some degree of bias in the rates of vancomycin utilization.

Our experience provides a valuable framework for implementing a successful TASP protocol in the community health system setting utilizing non-ID trained pharmacists and rapid diagnostics to achieve meaningful stewardship goals.

## Supporting information

Trzebucki et al. supplementary material 1 Trzebucki et al. supplementary material

Trzebucki et al. supplementary material 2 Trzebucki et al. supplementary material

## Supplementary material

The supplementary material for this article can be found at https://doi.org/10.1017/ash.2024.43.
