# Supplementary figures and images for: Impact of a pharmacy-driven MRSA nares screening protocol on vancomycin discontinuation in a tele-antimicrobial stewardship model
Source: Antimicrob Steward Healthc Epidemiol. 2024 Apr 22;4(1):e56. doi: 10.1017/ash.2024.43 (PMC11036443; doi:10.1017/ash.2024.43)

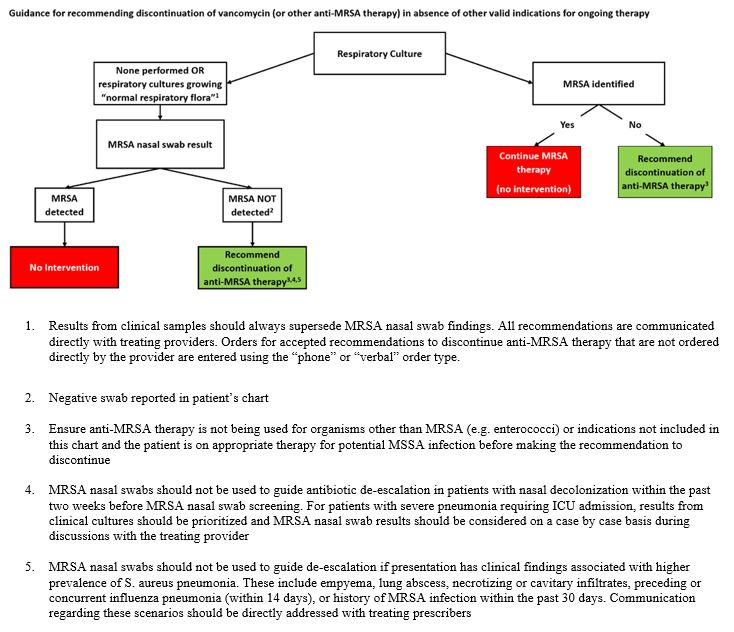

Supplement: Trzebucki et al. supplementary material 1 — Trzebucki et al. supplementary material [file S2732494X24000433sup001.tiff]
