# Supplementary material for: Impact of a pharmacy-driven MRSA nares screening protocol on vancomycin discontinuation in a tele-antimicrobial stewardship model
Source: Antimicrob Steward Healthc Epidemiol. 2024 Apr 22;4(1):e56. doi: 10.1017/ash.2024.43 (PMC11036443; doi:10.1017/ash.2024.43)
Supplement: Trzebucki et al. supplementary material 2 — Trzebucki et al. supplementary material [file S2732494X24000433sup002.docx]

Supplemental Table 2 – Test performance characteristics for samples with simultaneous respiratory culture and nasal MRSA testing performed

| Pre-protocol PPV/NPV | 87 cases with both MRSA nares testing and respiratory culture completed | | |
| --- | --- | --- | --- |
|  | Disease (resp culture) + | Disease (resp culture) - | PPV/NPV |
| Test (nares) + | 2 | 8 | **20.00%** |
| Test (nares) - | 3 | 70 | **95.89%** |
| Sn/Sp | 40.00% | 89.74% |  |
|  | | | |
| Post-protocol PPV/NPV | 80 cases with both MRSA nares testing and respiratory culture completed | | |
|  | Disease (resp culture) + | Disease (resp culture) - | PPV/NPV |
| Test (nares) + | 3 | 5 | **37.50%** |
| Test (nares) - | 3 | 69 | **95.83%** |
| Sn/Sp | 50.00% | 93.24% |  |
